# Neurological Manifestation in Hospitalized Patients With Acute SARS-CoV-2 Infection

**DOI:** 10.7759/cureus.44598

**Published:** 2023-09-03

**Authors:** Natasa Pejanovic-Skobic, Kristina Galic, Ilijana Kapcevic, Svjetlana Grgic, Marina Vasilj, Sandra Lakicevic, Marija Bender, Tanja Zovko

**Affiliations:** 1 Clinic of Neurology, University Clinical Hospital Mostar, Mostar, BIH; 2 Department of Pulmonary Diseases and Tuberculosis, University Clinical Hospital Mostar, Mostar, BIH; 3 Emergency Department, Health Care Center Rama, Rama-Prozor, BIH; 4 Clinic for Infectious Diseases, University Clinical Hospital Mostar, Mostar, BIH

**Keywords:** comorbidities and covid-19, loss of taste, loss of smell, cerebrovascular incident, neurological manifestations, sars-cov-2 infection

## Abstract

Objective: The main objective of this research is to determine the prevalence and characteristics of neurological manifestations in hospitalized patients with SARS-CoV-2 infection.

Methods: A cross-sectional study was conducted. 572 hospitalized patients at the COVID Department of Pulmonology of the Mostar University Clinical Hospital in the six-month period from October 31, 2020, to April 30, 2021, were included. We analyzed the incidence of neurological manifestations and the influence of comorbidities and metabolic syndrome on stroke incidence in COVID-19 patients. We analyzed hospital length of stay and mortality in patients with and without neurological manifestations. The research was conducted with respect to all the determinants of the Helsinki Declaration.

Results: 572 patients, 351 men (61.4%), and 221 women (38.6%) were included. A fatal outcome was present in a quarter of patients (25.3%). Neurological manifestations were found in 163 patients (28.5%). Myalgia was the most common (16.1%). The following were reported: headache (9.6%), loss of taste (7.34%), loss of smell (6.8%), and vertigo (2.5%). There was a significant difference regarding loss of smell between males and females (p=0.04). The cerebrovascular incident was present in 2.97% of patients and was more frequent in the group of patients with metabolic syndrome. Patients with neurological manifestations had a longer hospital stay, but it was not statistically significant (p=0.9319). The presence of neurological manifestations in general did not influence the mortality rate.

Conclusion: Patients with SARS-CoV-2 infection can present with neurologic findings such as myalgia, headache, loss of smell or taste, vertigo, as well as cerebrovascular incidents. Patients with neurological manifestations had longer hospital stays, but the presence of neurological manifestations in general did not influence the mortality rate.

## Introduction

Coronavirus disease 2019 (COVID-19) is an acute respiratory disease caused by a new coronavirus called SARS-CoV-2. It was first reported at the end of December 2019 in Wuhan, China, in the form of several cases of pneumonia of unknown etiology, and has since spread throughout the world [1].

Among other symptoms, it has been shown that SARS-CoV-2 infection leads to various neurological manifestations [2]. Numerous neurological disorders can occur as part of the COVID-19 disease, affecting the central and peripheral nervous systems. According to research carried out in Wuhan, China, different neurological manifestations were found in patients with COVID-19 [3]. In patients with a milder clinical form, neurological symptoms are mostly limited to non-specific disorders such as headache, dizziness, anosmia, and ageusia, while in critically ill patients, more serious complications occur with a significantly higher mortality rate [4]. The occurrence of long-term neurological complications in terms of acceleration or initiation of neurodegenerative diseases is of concern [5]. While COVID-19 was initially viewed as a pulmonary disease, it is now considered a multisystemic disease in which neurological symptoms and syndromes may be prominent manifestations. In acutely ill patients, COVID-19 may involve neurological complications with implications for recovery from the disease and long-term morbidity, and in the outpatient setting, permanent neurological symptoms may manifest as part of the post-acute consequences of SARS-CoV-2 infection (neuro-PASC) [6].

Myalgias, headaches, encephalopathy, dizziness, anosmia, and dysgeusia together make up 95.8% of neurological manifestations at the beginning of COVID-19 and 91.4% of manifestations during acute COVID-19 [7]. While myalgias, headaches, and anosmia/dysgeusia may be uncomfortable for the patient, in the experience of some investigators, they have not affected the course of the acute illness, mortality, or the occurrence of severe disability. On the other hand, some of the neurological complications of COVID-19, such as cerebrovascular diseases in hospitalized patients, can affect mortality or significant morbidity [7]. Most patients with COVID-19 have mild respiratory illnesses such as a dry cough, fever, and dyspnea. However, various neurological manifestations are also associated with COVID-19 at the time of onset or during the illness [3]. Based on the available literature, patients with a severe COVID-19 infection tend to develop more neurological abnormalities compared to those with a mild infection [8]. Patients with mild illness have any of the various signs and symptoms of COVID-19 but do not have shortness of breath, dyspnea, or abnormal chest imaging. Patients with severe illness have decreased oxygen saturation measured by pulse oximetry in room air, an impaired ratio of arterial partial pressure of oxygen to the fraction of inspired oxygen, and an increased respiratory rate [9].

Our study analyzed neurological manifestations in severe and moderate COVID-19 infection in hospitalized patients and the effect of neurological manifestations on the length of hospital stay and the outcome of acute SARS-CoV-2 infection.

## Materials and methods

Patients who were treated at the COVID hospital of the Pulmonology Department of the Mostar University Clinical Hospital in the six-month period from October 31, 2020, to April 30, 2021 and fit the inclusion criteria were included in the research. The total number of patients is 572, of which 351 are men and 221 are women.

A cross-sectional study was conducted. Patient information was collected from the Hospital Information System (HIS) based on medical documentation in the form of a medical history. Before being admitted to the COVID-19 ward, the patients had an acute infection with the coronavirus (COVID-19), confirmed by PCR. Hospitalized patients aged over 18 with severe unstable, but not critical, COVID-19 disease (MEWS 3-4) and moderately severe stable COVID-19 disease (MEWS ≤ 3) with comorbidity, according to the guidelines for the treatment of COVID-19 patients issued by the World Health Organization (WHO) in 2020, were included. Severe unstable but not critical illness (MEWS 3-4) was defined as illness in patients with clinical or laboratory signs of impaired ratio of arterial partial pressure of oxygen to fraction of inspired oxygen, with dyspnea, tachypnea, breathing shortness, and the need for oxygen supplementation >4 L/min for regaining oxygen saturation measured by pulse oximetry above 92%, but without critical signs (acute respiratory failure, septic shock, impairment of consciousness) [9].

Exclusion criteria were: age under 18, a mild form of COVID-19 infection, and patients with confirmed COVID-19 infection who were not hospitalized.

The study was based on clinical manifestations and not on pathophysiological phenomena. Neurological symptoms and signs were confirmed by neurological examination during hospitalization. For each subject, the following were analyzed: demographic characteristics (age, sex), the most common comorbidities (arterial hypertension, diabetes, metabolic syndrome), the influence of coagulation factors on the occurrence of neurological manifestations, qualitative disorders of the state of consciousness (confusion, delirium), the occurrence of ischemic and hemorrhagic cerebrovascular insults, epileptic seizures, myalgia, dizziness, headache, ageusia, anosmia, the number of hospital days in patients with and without neurological manifestations, and the influence of the presence of non-neurological manifestations on the outcome of treatment. Metabolic syndrome was defined as the clustering of at least three of the following five medical conditions: abdominal obesity, high blood pressure, high blood sugar, high serum triglycerides, and low serum high-density lipoprotein (HDL) [10].

The software systems IBM SPSS Statistics for Windows, Version 25.0 (Armonk, NY: IBM Corp.), and Microsoft Excel 2016 (Microsoft® Corp., Redmond, WA) were used for statistical analysis. The tests used are the χ2 test, Mann-Whitney U test, Fisher's exact test, Kruskal-Wallis test, Student's t-test, ANOVA test, and normality test (Kolmogorov-Smirnov, Shapiro-Wilk test). A p-value of <0.05 is considered statistically significant.

The research and all protocols were approved by the Ethics Committee of the Faculty of Medicine of the University in Mostar (approval number 1175/22). All procedures performed followed the institutional ethical standards of the research committee and the Declaration of Helsinki [11].

## Results

Demographic data show that 572 patients participated in the study, of whom 351 were men (61.4%) and 221 were women (38.6%). The median age for men was 67 years, and for women, it was 72 years. The oldest patient was 96 years old, and the youngest was 23 years old (Table 1).

**Table 1:** Demographic data of respondents

	Male	Female
N (%)	351 (61.4)	221 (38.6)
x͂ (years)	67	72
IQR (years)	20.75	20
Min (years)	23	23
Max (years)	96	96

The total number of patients who recovered is 427 (74.7%), and the total number of patients with a fatal outcome is 145 (25.3%) (Table 2).

**Table 2:** Recovery and death rate

Outcome	N	%
Recovery	427	74.7
Death	145	25.3

**Figure 1:**
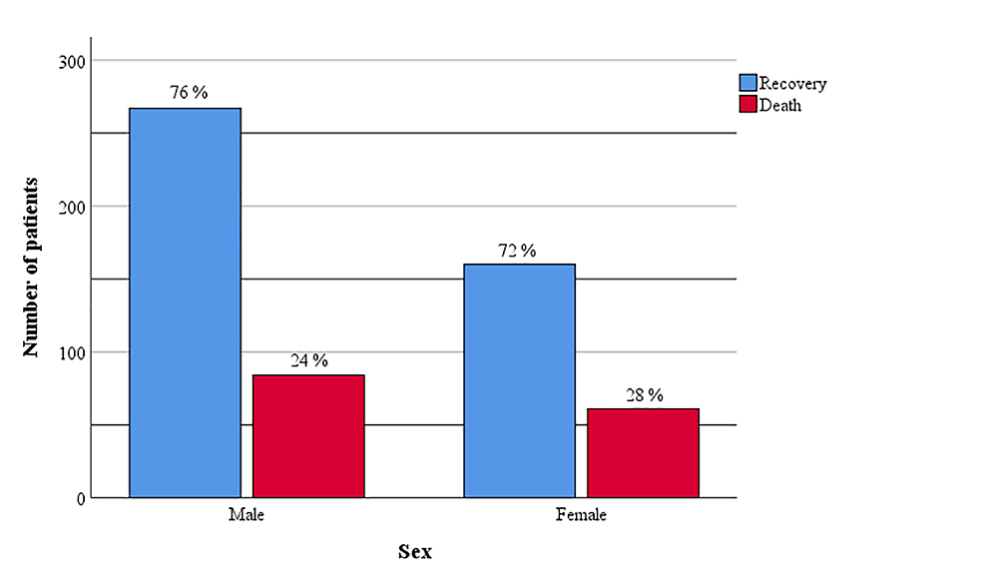
Gender distribution of recovered and deceased patients

Of the total number of men (N=351), 76% recovered, and 24% had a fatal outcome. Of the total number of women (N=221), 72% recovered, and 28% had a fatal outcome (Figure 1).

**Figure 2:**
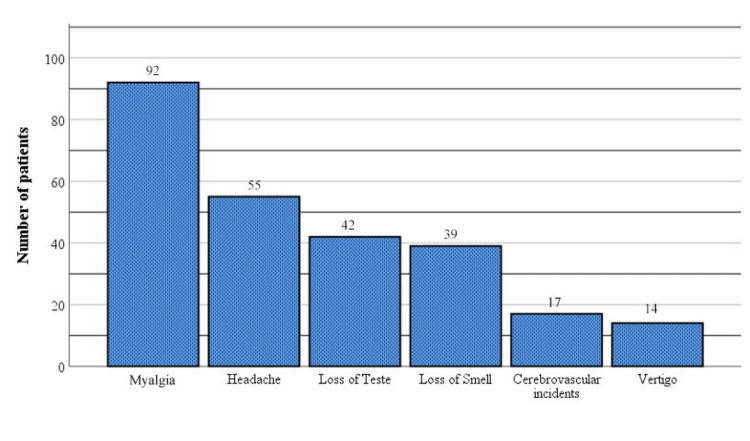
Neurological and neuromuscular manifestations in patients with COVID-19 infection

Myalgia is the most common neuromuscular manifestation and occurred in 92 patients (16.1%). Headache was present in 55 patients (9.6%), loss of taste in 42 patients (7.34%), loss of smell in 39 patients (6.8%), and the least number of patients had vertigo/dizziness, which occurred in 14 of them (2.5%). A cerebrovascular incident/stroke was found in 17 patients (2.97%) (Figure 2).

**Figure 3:**
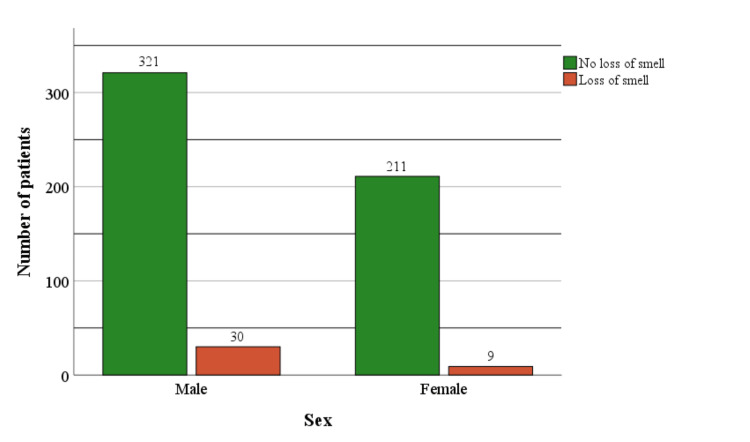
Loss of smell in patients with COVID-19 infection

There are statistically significant differences in the frequency of neurological manifestations in relation to gender when it comes to loss of smell and taste. Men had a more frequent symptom of loss of smell. 30 (8.5%) of the male subjects, in contrast to 9 (4.1%) of the female subjects, recorded a loss or disorder of the sense of smell, making up 6.8% of the subjects with this neurological manifestation (χ2= 4.22, p=0.040) (Figure 3).

**Figure 4:**
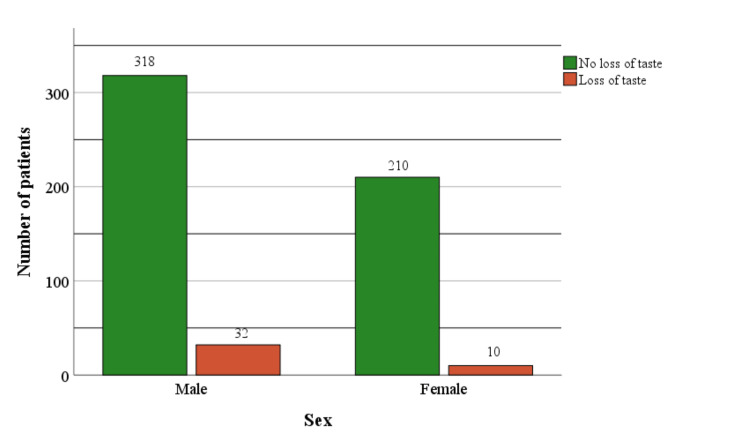
Loss of taste in patients with COVID-19 infection

As the senses of smell and taste are connected, men also had a statistically significantly higher incidence of sensory loss compared to women. Thirty-two male subjects (9.1%) and ten female subjects (4.5%) had a loss of sense of taste (χ2 = 4.183, p=0.041) (Figure 4).

Metabolic syndrome was present in 113 patients (19.8%). The cerebrovascular incident was more frequent in the group of patients with metabolic syndrome (15.04%) compared to the group without metabolic syndrome (2.61%), but the difference was not statistically significant (Fisher exact test, p=0.3502) (Table 3).

**Table 3:** Metabolic syndrome and cerebrovascular incident in COVID 19 patients

		Cerebrovascular incident		Total
		NO	YES	
Metabolic syndrome	N0	447	12	459
	YES	108	5	113
Total		555	17	572

The Chi-square test determined that there is no statistically significant association between the outcome of recovery or death and the occurrence of neurological manifestations. Out of a total of 163 patients with neurological manifestations, 28 of them had a fatal outcome (17.2%), while in the group of patients without neurological manifestations, out of a total of 409, the fatal outcome was recorded in 117 patients (28.6%). In the group of patients without neurological manifestations, the mortality rate was higher (χ2=8.044, p=0.005). The presence of neurological manifestations generally did not affect the mortality rate.

There was no significant association between disturbed coagulation factors D-dimers (p=0.325), INR (p=0.56), and platelet number (p=0.819) and the occurrence of a stroke.

Patients with neurological manifestations had a longer hospital stay (10 days) compared to patients without neurological manifestations (9 days), but this difference in length of hospital stay is not statistically significant (Mann-Whitney U test, p=0.9319).

## Discussion

In this cross-sectional study of 572 hospitalized patients with severe and moderate infections with COVID-19, the presence of neurological manifestations and their impact on mortality and hospital stay were analyzed.

SARS-CoV-2 infection is associated with several neurological symptoms and manifestations due to involvement of the central nervous system (CNS) and peripheral nervous system (PNS), ranging from non-specific symptoms (headache, myalgia, dizziness, fatigue) to cerebrovascular disease, encephalitis, movement disorders, myelitis, and cranial and peripheral neuropathies [12].

Of these, 163 (28.5%) had various neurological manifestations involving the CNS, PNS, and skeletal muscles. These results are consistent with the results of previous studies. According to some earlier studies, more than 35% of patients with COVID-19 develop neurological symptoms. Some patients with COVID-19 may have neurological symptoms as the initial presentation of the disease [13]. However, the prevalence of neurological signs and symptoms is higher in patients with a severe COVID-19 infection, which may be a consequence of cerebral hypoxia due to respiratory failure [14].

Myalgia was the most common neuromuscular finding (16%), which is consistent with the results of previous research [15].

Headaches were present in 9.6% of patients. Other studies have reported a prevalence of headaches ranging from 6.5 to 23%, with an average prevalence of 8% [16].

Loss of smell was recorded in 6.8% and loss of taste in 7.34% of patients. One study reported impairment of smell and taste in 5.1% and 5.6% of patients [3], which is consistent with our results. However, numerous studies concerning taste and smell disorders have also been published, with large variations in reported frequency. Loss of smell and taste were reported more frequently in patients with mild or moderate COVID-19 compared to severe or critical disease courses [17]. Ahmed et al. reported that confirmed COVID-19 female cases were five times more likely to experience a loss of smell or taste than males [18]. This is an interesting result because it is the opposite of our results. There is no definite explanation for these gender differences. Maybe some future studies regarding population and gender-specific behaviors, genetic or hormonal factors, sex differences in biological pathways related to SARS-CoV-2 infection, and the underlying mechanisms will give possible explanations for these observed gender differences.

The cerebrovascular incident was present in 17 patients (2.97%). Our results are in accordance with results from other studies. Mao and associates report that 2.8% of patients developed acute cerebrovascular diseases [3]. We analyzed the association of metabolic syndrome with cerebrovascular incidents in the case of COVID-19 infection. The cerebrovascular incident was statistically significantly increased in the group of patients with metabolic syndrome (15.04%) compared to the group without metabolic syndrome (2.61%). Other studies show that patients who developed acute cerebrovascular events were more likely to have cardiovascular risk factors, including hypertension, diabetes, and a history of other diseases with previous cardiovascular or cerebrovascular disease [19].

Vertigo was present in 2.5% of our patients. Most studies did not distinguish between the terms instability and dizziness. Gallus et al. reported a prevalence of unsteadiness of 8.3% and a prevalence of vertigo of 2% [20].

The presence of neurological manifestations was associated with a longer hospital stay, but this prolongation of the hospital stay was not statistically significant. Liotta et al. also reported that patients with neurological manifestations had longer hospitalizations [7].

Our results show that the presence of neurological manifestations generally does not affect the mortality rate from COVID-19 infection. Given that the most common neurological manifestations were myalgia, headache, and loss of smell and taste, they are not associated with a worse outcome. Our results are consistent with the results of the international cohort study by Porta-Etessam et al., which showed that the presence of the most common neurological manifestations, loss of smell and taste, is associated with a better prognosis and is inversely proportional to death outcome in patients with COVID-19 infection [21].

If we talk about the commonest symptoms, our results are consistent with the results of the recent study by Beghi et al. They also found that the most common symptoms were headache, followed by hyposmia/hypogeusia, myalgia, and vertigo [22].

The most common symptoms were headache (41.4%), followed by hyposmia/hypogeusia (31.5%), myalgia (30.1%), vertigo (21.6%), sleep-wake disturbances (including sleepiness/hypersomnia; 16.4%), and ataxia (8.8%). Cognitive dysfunction (29.5%), stroke (25.7%), dysautonomia (14.7%), peripheral neuropathies (9.5%), movement disorders (9.3%), and seizures (including status epilepticus; 8.3%) were the predominant clinical diagnoses.

It is interesting that we did not identify any cases of Guillain-Barre syndrome in our patients hospitalized with acute SARS-CoV-2 infection, but we also found some studies like that of Liotta et al. [7]. who also did not find any patients with this neurological manifestation.

Despite many studies, the exact rate of neurological manifestations in patients with COVID-19 remains unclear. An explanation for the large difference between the rates of neurological manifestations in patients with COVID-19 in different studies could be the fact that different reporting standards are used. Also, different case definitions of neurological manifestations that sometimes use a pathophysiology-based approach and sometimes a symptom-based approach may be one of the reasons for such diversity in study results.

There are some limitations to this study. First, we included only hospitalized patients. Symptoms such as loss of smell and taste and dysfunction of the olfactory and gustatory systems were determined only by the patient, who reported them himself, without an objective assessment by any otorhinolaryngologist. Since this study was conducted at a single tertiary center, the spectrum of neurological complications may not be fully represented. The study did not assess temporal patterns of neurological symptoms, so we do not know which symptoms may have appeared first and in what order. Despite these limitations, we present the first study on neurological symptoms in hospitalized patients with severe and moderate forms of COVID-19 in Bosnia and Herzegovina.

## Conclusions

We presented adult hospitalized patients with new-onset neurologic manifestations associated with the SARS-CoV-2 infection. The most common neurological findings in those patients were myalgia, headache, loss of smell or taste, vertigo/dizziness, as well as a cerebrovascular incident/stroke. Patients with neurological manifestations had longer hospital stays, but the presence of neurological manifestations in general did not influence the mortality rate. It is important to raise awareness of the presence of neurological manifestations in patients with SARS-CoV-2 infection so that they can be recognized in time and adequately treated.

## References

[1] Tesini BL. COVID-19 (2023). COVID-19: MSD Manual Consumer Version. MSD MANUAL Consumer Version.

[2] Chen T, Wu D, Chen H (2020). Clinical characteristics of 113 deceased patients with coronavirus disease 2019: retrospective study. BMJ.

[3] Mao L, Jin H, Wang M (2020). Neurologic manifestations of hospitalized patients with coronavirus disease 2019 in Wuhan, China. JAMA Neurol.

[4] Keyhanian K, Umeton RP, Mohit B, Davoudi V, Hajighasemi F, Ghasemi M (2020). SARS-CoV-2 and nervous system: from pathogenesis to clinical manifestation. J Neuroimmunol.

[5] Iadecola C, Anrather J, Kamel H (2020). Effects of COVID-19 on the nervous system. Cell.

[6] Graham EL, Koralnik IJ, Liotta EM (2022). Therapeutic approaches to the neurologic manifestations of COVID-19. Neurotherapeutics.

[7] Liotta EM, Batra A, Clark JR, Shlobin NA, Hoffman SC, Orban ZS, Koralnik IJ (2020). Frequent neurologic manifestations and encephalopathy-associated morbidity in Covid-19 patients. Ann Clin Transl Neurol.

[8] Ahmed MU, Hanif M, Ali MJ (2020). Neurological manifestations of COVID-19 (SARS-CoV-2): a review. Front Neurol.

[9] (2023). WHO COVID-19: case definitions. https://apps.who.int/iris/bitstream/handle/10665/360579/WHO-2019-nCoV-Surveillance-Case-Definition-2022.1-eng.pdf.

[10] (2023). Metabolic syndrome. https://www.mayoclinic.org/diseases-conditions/metabolic-syndrome/symptoms-causes/syc-20351916.

[11] (2013). World Medical Association Declaration of Helsinki: ethical principles for medical research involving human subjects. JAMA.

[12] Moro E, Priori A, Beghi E (2020). The international European Academy of Neurology survey on neurological symptoms in patients with COVID-19 infection. Eur J Neurol.

[13] Jiang F, Deng L, Zhang L, Cai Y, Cheung CW, Xia Z (2020). Review of the clinical characteristics of coronavirus disease 2019 (COVID-19). J Gen Intern Med.

[14] Azhideh A (2020). COVID-19 neurological manifestations. Int Clin Neurosci J.

[15] Leven Y, Bösel J (2021). Neurological manifestations of COVID-19: an approach to categories of pathology. Neurol Res Pract.

[16] Rodriguez-Morales AJ, Cardona-Ospina JA, Gutiérrez-Ocampo E (2020). Clinical, laboratory and imaging features of COVID-19: a systematic review and meta-analysis. Travel Med Infect Dis.

[17] Chen X, Laurent S, Onur OA, Kleineberg NN, Fink GR, Schweitzer F, Warnke C (2021). A systematic review of neurological symptoms and complications of COVID-19. J Neurol.

[18] Ahmed MM, Sayed AM, El Abd D, Fares S, Said MS, Elsayed Sedik Ebrahim E (2021). Gender difference in perceived symptoms and laboratory investigations in suspected and confirmed COVID-19 cases: a retrospective study. J Prim Care Community Health.

[19] Li Y, Li M, Wang M (2020). Acute cerebrovascular disease following COVID-19: a single center, retrospective, observational study. Stroke Vasc Neurol.

[20] Gallus R, Melis A, Rizzo D (2021). Audiovestibular symptoms and sequelae in COVID-19 patients. J Vestib Res.

[21] Porta-Etessam J, Núñez-Gil IJ, González García N (2021). COVID-19 anosmia and gustatory symptoms as a prognosis factor: a subanalysis of the HOPE COVID-19 (Health Outcome Predictive Evaluation for COVID-19) registry. Infection.

[22] Beghi E, Moro E, Davidescu EI (2023). Comparative features and outcomes of major neurological complications of COVID-19. Eur J Neurol.

